# Interações e conversas em visitas de famílias à exposição “Ciencia del amor y del perdón”, no Maloka, em Bogotá, Colômbia

**DOI:** 10.1590/S0104-59702025000100024

**Published:** 2025-05-23

**Authors:** Alice Ribeiro, Juliana Araujo, Luisa Massarani, Rafael Velloso Luz, Grazielle Scalfi, Sigrid Falla

**Affiliations:** i Pesquisadora, Instituto Nacional de Comunicação Pública da Ciência e Tecnologia/Fiocruz; Casa de Oswaldo Cruz/Fiocruz. Rio de Janeiro – RJ – Brasil alice.ribeiro.geo@gmail.com; ii Pesquisadora, Instituto Nacional de Comunicação Pública da Ciência e Tecnologia/Fiocruz; Casa de Oswaldo Cruz/Fiocruz. Rio de Janeiro – RJ – Brasil dearaujojm@gmail.com; iii Coordenadora, Instituto Nacional de Comunicação Pública da Ciência e Tecnologia/Fiocruz; Casa de Oswaldo Cruz/Fiocruz. Rio de Janeiro – RJ – Brasil luisa.massarani@fiocruz.br; iv Doutorando, Universidade do Estado do Rio de Janeiro. Rio de Janeiro – RJ – Brasil velloso.rafa@gmail.com; v Pesquisadora, Instituto Nacional de Comunicação Pública da Ciência e Tecnologia/Fiocruz; Casa de Oswaldo Cruz/Fiocruz. Rio de Janeiro – RJ – Brasil graziscalfi@gmail.com; vi Diretora de arquitetura de experiências, Centro de Ciências Maloka. Bogotá – Colômbia sfalla@maloka.org

**Keywords:** Museus e centros de ciências interativos, Público familiar, Interatividade, Mediação em museus, Interactive museums and science centers, Family visitors, Interactivity, Mediation in museums

## Abstract

O artigo analisa interações e conversas de dez famílias em visita à exposição “Ciencia del amor y del perdón”, do museu Maloka – em Bogotá, Colômbia –, que apresenta emoções sob perspectivas neurocientíficas e das ciências humanas e explora ferramentas da psicologia para o perdão, em um contexto de “pós-conflito”, após assinatura de acordo de paz entre o governo e o maior grupo guerrilheiro colombiano. Os dados, coletados em dezembro de 2022, por meio de gravações das visitas em câmera GoPro acoplada ao tórax de uma criança de cada grupo, foram analisados com uso do *software* Dedoose. Os resultados indicaram que a mediação dos adultos e a proposta expográfica possibilitaram interatividade física e mental.

## Abstract (en)

This article analyzes the interactions and conversations of ten families visiting the exhibition “The science of love and forgiveness”, at Maloka, in Bogotá, Colombia, which explores emotions from a neuroscientific and human sciences perspective, as well as psychological approaches to forgiveness in a post-conflict context, namely, since the peace agreement between the government and Colombia’s largest guerrilla group. Family visits in December 2022 were recorded using GoPro cameras attached to the chest of a child from each group. The data were analyzed using Dedoose software. The mediation of adults and the exhibition design were found to foster physical and mental interaction.

Em 2016, o governo colombiano e o grupo guerrilheiro Fuerzas Armadas Revolucionarias de Colombia-Ejército del Pueblo (Farc-EP) firmaram o *Acuerdo final para terminación del conflicto y la construcción de una paz estable y duradera* (Acordo de Havana) (Colombia, 2016). A assinatura do acordo visou à resolução do conflito armado que se estende por mais de trinta anos no país, já tendo resultado no desaparecimento forçado de mais de 57.200 pessoas e no deslocamento de 5,2 milhões de pessoas do interior para outras regiões (principalmente para a capital do país, Bogotá), como forma de escapar da violência e da pobreza (Vasconcellos, 2023). Entretanto, um mês após a assinatura, e “contrariando todas as expectativas, 50,2% da população votou contra a sua ratificação em referendo”, colocando “em evidência a polarização ideológica da sociedade colombiana” (Marín, Alzate, 2020, p.2).

Nesse contexto, o Maloka, museu de ciências localizado em Bogotá, tem realizado um debate sobre a necessidade de construir um ambiente de paz na vida cotidiana do país, compreendendo a reconstrução social como uma estratégia realizada de baixo para cima, que abarca todos os setores sociais e exige grandes mudanças coletivas (Alzate et al., 2018; Galtung, 1996). Aplicada à divulgação científica, trata-se de uma perspectiva que considera que a ciência e sua representação são atravessadas por dinâmicas de poder (Macdonald, 1997; Macdonald, Silverstone, 1992; Marandino, Contier, 2017).

A exposição “Ciencia del amor y del perdón” [Ciência do amor e do perdão], especificamente, inaugurada em 20 de fevereiro de 2019, busca promover a reflexão sobre o papel da gestão das emoções nas relações cotidianas e sobre os benefícios do perdão para a saúde física e mental (Luskin, 2002). Assim, disponibiliza ferramentas das neurociências, da psicologia e de outras ciências humanas e sociais para que as pessoas possam compreender melhor as emoções (suas e dos outros), a fim de promover empatia com aqueles considerados diferentes e avançar para um processo de reconciliação em diferentes espaços da vida cotidiana. Ainda que não aborde diretamente o conflito armado, o processo de paz e os argumentos que polarizam a opinião pública sobre esses temas, inclui algumas situações sensíveis sobre os efeitos de diversas violências na vida das pessoas, para que os públicos possam conversar abertamente sobre elas.

Há de se mencionar que, na América Latina, região com um passado marcado por ditaduras cívico-militares e um presente atravessado por outros conflitos violentos, surgiram, ao longo da segunda metade dos anos 2000 e de toda a década de 2010, museus que tematizam as memórias traumáticas associadas a essas experiências (Giroto, 2021).^1^ Podemos dizer que a exposição analisada neste artigo se insere nesse contexto latino no qual as violências do passado e do presente ganham cada vez mais destaque no campo museal. Por outro lado, enquanto esses museus surgiram especificamente para abordar essas memórias traumáticas, o Maloka é um museu de ciências interativo surgido há mais tempo (final dos anos 1990) e que busca, na atualidade, pautar temáticas socialmente relevantes transversalmente às suas exposições.

Diante dessas especificidades e da relevância da exposição no momento atual colombiano, bem como da importância do Maloka como um museu de ciências interativo que objetiva a apropriação social do conhecimento, os pesquisadores do Instituto Nacional de Comunicação Pública da Ciência e da Tecnologia (INCT-CPCT/Fiocruz) optaram por investigar a experiência de visita de famílias à exposição, no âmbito do projeto de pesquisa “Olhar do visitante”, que investiga interações e conversas ocorridas em visitas de grupos a centros e museus de ciências.^2^ Assim, neste estudo, observamos as visitas de dez grupos familiares à exposição “Ciencia del amor y del perdón”. Nosso objetivo é identificar as interações e conversas mais relevantes, analisar como elas ocorrem no âmbito familiar e refletir sobre as interações entre os diferentes atores da visita (educadores, crianças e familiares adultos) e deles com a exposição (incluindo seus aparatos interativos e textos).

Adotamos a perspectiva sociocultural de aprendizagem (Vygotsky, 1978), que, aplicada ao campo dos museus, tem estimulado, desde a década de 1980, estudos que exploram os diálogos e interações ocorridos durante a visita museal, envolvendo visitantes, espaço museal, objetos e profissionais do museu. Alguns autores que realizam esse tipo de estudo são Callanan, Cervantes, Loomis (2011); Crowley et al. (2001); Crowley, Pierroux, Knutson (2014); Ellenbogen, Luke, Dierking (2007); Gaspar (1993); Massarani et al. (2019b, 2020, 2021a, 2021b, 2022a); McClain, Zimmerman (2014, 2016, 2019); Rocha et al. (2021); Rodrigues, Afonso (2015); Sápiras (2007); e Shaby, Assaraf, Tal (2019).

Nessa abordagem, entende-se que o aprendizado não ocorre apenas em nível individual, mas também social, sendo influenciado por diversos fatores, como o espaço do museu, os interesses e conhecimentos prévios, os aspectos sociais e as interações entre os sujeitos. Assim, diferentes autores argumentam que o aprendizado durante as visitas ocorre no âmbito de interações sociais colaborativas (Ash, 2003; Callanan, Cervantes, Loomis, 2011; Ellenbogen, 2002; Shaby, Assaraf, Tal, 2019).

As famílias, que, em geral, buscam na visita museal uma oportunidade de passarem tempo juntos, em um momento de lazer e aprendizado (Crowley, Pierroux, Knutson, 2014), têm despertado o interesse dos pesquisadores da área. Além de serem um importante público dos museus (Crowley, Pierroux, Knutson, 2014), os grupos familiares, constituídos por pessoas de diferentes gerações que compartilham um *background* em comum (Ellenbogen, 2002), costumam ter suas visitas marcadas por interações sociais por meio das quais educadores e/ou responsáveis fazem a mediação social do aprendizado das crianças (Ash, 2003; Cazelli, Marandino, Studart, 2003; Crowley, Pierroux, Knutson, 2014; Jant et al., 2014; Pattison, Rubin, Wright, 2017; Pavão, Leitão, 2007; Rodari, Merzagora, 2007; Studart, 2005). Assim, sendo a linguagem a ferramenta de mediação entre diferentes sujeitos e entre eles e os objetos, a interação social e o diálogo passam a ter um papel central na coconstrução do conhecimento e no aprendizado (Ash, 2003; Shaby, Assaraf, Tal, 2019).

Além das interações entre os sujeitos, outro fator que influencia a fruição e o aprendizado é o *design* expositivo. Nessa perspectiva, a interatividade tem sido considerada um importante pilar para aproximar os museus e centros de ciências do cotidiano do público não especializado (McManus, 1992). Ainda que inicialmente pensada em sua dimensão física, ou seja, a partir da possibilidade de tocar e manusear objetos, atualmente, o conceito é compreendido em sentido mais amplo, com base em Wagensberg (2005, p.311), que defende uma “museografia com objetos reais, mas capaz de expressá-los de uma forma triplamente interativa: interatividade manual (‘*hands on*’ no jargão museal atual), interatividade mental (‘*mind on*’) e interatividade cultural ou emocional (‘*hearts on*’)”.^3^

Essa ampliação do conceito insere-se em um contexto de defesa de museus de ciências participativos, interessados nas questões sociais que afetam seus públicos e na promoção de sua autonomia no que concerne à elaboração de experiências museais individualizadas (Padilla, 2001; Paula, 2017). Trata-se, portanto, de um conceito interessante para pensar a exposição aqui abordada, uma vez que ela está mergulhada em um contexto social delicado e tangencia questões sociais sensíveis, diante das quais se torna pertinente o amplo debate social, em prol de diálogos saudáveis e conciliatórios.

## A pesquisa: espaço, dados, resultados

### Local de estudo

A exposição “Ciencia del amor y del perdón”^4^ tem duas partes principais. A primeira descreve emoções e versa sobre a importância de geri-las adequadamente. Inicialmente, aborda as emoções dos animais como um processo evolutivo; em seguida, apresenta as emoções básicas (ira, medo, tristeza, alegria e indignação). Cada uma das estações traz uma proposta interativa que evoca a sensação da emoção abordada, um painel com uma explicação neuroquímica e uma atividade que convida o visitante a expressar as situações que lhe causam tal emoção. As estações relativas a essa parte da exposição são:

1. “¿Los animales sienten emociones?” [Os animais sentem emoções?]: trata das emoções sentidas por animais. A partir de uma tela sensível ao toque, apresenta exemplos de manifestações de tristeza e alegria em mamíferos. A tela traz ainda uma espécie de história em quadrinhos que narra uma situação fictícia de um orangotango salvando um passarinho que caiu na água.
2. “La ira es un fuego interior que nos devora” [A ira é um fogo interior que nos devora]: aborda de que maneira a ira mexe com nosso corpo e como situações distintas podem nos suscitar raiva. O visitante é convidado a gritar em orifícios para extravasar sua ira. Há marcadores luminosos que se movimentam, demonstrando quão alto foi o grito. Outro totem consiste em tubos com frases de situações consideradas desagradáveis. Os visitantes são convidados a colocar pedras nos tubos relativos a situações que lhes causam incômodo.
3. “La tristeza acerca los otros” [A tristeza nos aproxima dos outros]: discute de que maneira a tristeza altera nosso corpo e como situações distintas podem nos deixar tristes. Possui uma *jukebox* com músicas famosas na Colômbia, geralmente de tom melancólico. Em outra atividade, os visitantes podem escrever em um papel adesivo algo que lhes causa tristeza e colar no painel.
4. “La indignación es una emoción que transforma” [A indignação é uma emoção que transforma]: constituída por uma tela sensível ao toque, com situações que poderiam gerar indignação, e cinco botões que, numerados de um a cinco, representam uma escala de pouco (botão de número um) a muito indignado (botão de número cinco). Ao ver cada imagem, os visitantes deveriam apertar um dos botões para indicar seu grau de indignação. Ao fim, é gerado um gráfico que mostra qual dos participantes mais se indignou.
5. “El miedo, emoción más antigua” [Medo, a emoção mais antiga]: uma espécie de cômodo escuro. Dentro dele, há janelas com tampas que, ao serem levantadas, dão acesso a textos narrando histórias de terror. Os grupos eram orientados a ler esses textos em uma determinada ordem e em voz alta. No texto da última janela, em vez de uma história de terror, há uma explicação da relevância do medo em nossas vidas. Passados alguns segundos com a tampa da última janela aberta, é emitido um som alto (simulando uma espécie de grito tenebroso) e uma luz no piso acende, causando uma sensação de “queda profunda no vazio”, a fim de assustar os visitantes. Em outra atividade, há uma tela sensível ao toque em que se pode escrever o nome de algo que lhe causa medo e inserir sons de instrumentos musicais, a fim de diminuir esse medo.
6. “La alegría le da sabor a la vida” [A alegria dá sabor à vida]: constituída por uma tela sensível ao toque acoplada a uma câmera e um fundo verde. Os visitantes podem tirar *selfies* e adicionar filtros nas fotos que simulam ambientes prazerosos diversos. Em seguida, podem enviá-las para seu próprio *e-mail*. Outra atividade faz alusão às “borboletas no estômago” causadas por algumas emoções. Nela, os visitantes aprendem a fazer uma borboleta de papel a partir de instruções disponíveis em um painel.

A segunda parte aborda a empatia, por meio de estações interativas, em que os visitantes são estimulados a se colocarem no lugar dos outros. As estações são:

1. “En los zapatos del otro” [No lugar do outro]: uma espécie de cabine telefônica. Ao atender o telefone, o visitante escuta pessoas narrando situações difíceis que viveram e como as superaram. No chão, existe uma espécie de sapato que o visitante coloca. À frente, há um espelho. A provocação é a de se colocar no lugar da pessoa que conta a história. Os casos abordam diferentes situações de discriminação, abuso ou escassez de recursos, alguns deles causados pelo conflito armado. Buscam demonstrar como as pessoas, com apoio e solidariedade, conseguem superar seus desafios, e, assim, gerar empatia sobre essas situações.
2. “El acoso escolar: un dolor de todos” [Assédio escolar: uma dor de todos]: aborda o problema do *bullying* escolar. A atividade consiste em um modelo de carteira escolar em que o visitante pode se sentar. Ao abrir o tampo da mesa, um áudio que simula violências escolares é reproduzido. O módulo convida os visitantes a sentar “na carteira do outro” e evoca as sensações tidas por uma pessoa em situação de *bullying*. Há ainda uma série de painéis que abordam possíveis indícios de que o assédio ou *bullying* escolar está ocorrendo e suas possíveis consequências, além de números de telefone para denúncia/auxílio.
3. “¿Abrazar es perdonar?” [Abraçar é perdoar?]: fala sobre os processos do perdão e o “poder” do abraço. Nele, os visitantes são convidados a abraçar ursos de pelúcias e uns aos outros. Intenciona abordar a empatia na aproximação com os outros.

Por fim, existe uma atividade de roleta de perguntas e desafios, por meio dos quais o visitante é convidado a realizar tarefas e refletir sobre as emoções abordadas na exposição.

A descrição das estações apresentada acima permite perceber que a exposição se caracteriza pela interatividade, pois, em todas elas, o público é convidado a realizar atividades que o estimulam não apenas a interagir fisicamente com os aparatos, como também, por meio disso, refletir sobre os conceitos das emoções abordadas e o modo como as vivenciam individualmente. Em algumas estações, essa reflexão é proposta por meio de imersão: o público se transporta para um ambiente mal assombrado, no qual sente medo; para uma conversa por telefone, na qual experimenta a empatia; para um ambiente escolar, no qual percebe como é sofrer *bullyin*g.

### Coleta e análise de dados

A coleta de dados ocorreu no dia 15 de dezembro de 2022 e teve como foco as famílias. Na entrada da exposição, as pesquisadoras^5^ abordavam grupos familiares de duas a cinco pessoas que continham pelo menos uma criança com idade entre 6 e 12 anos e os convidavam a participar da pesquisa. Eram explicados os objetivos e procedimentos metodológicos, que consistiam em duas etapas. Primeiro, era aplicado um questionário com o intuito de traçar o perfil do grupo e compreender seus hábitos culturais e de visitação a museus e centros de ciência. Em seguida, uma das crianças colocava uma câmera GoPro na cabeça ou no peito, e a família faria a visita sem interferências das pesquisadoras. Quando o grupo aceitava participar, era fornecido o “Termo de consentimento livre e esclarecido” para que um dos responsáveis adultos lesse e assinasse, e em seguida era iniciada a coleta de dados. Ao final da visita, o grupo devolvia a câmera e as pesquisadoras perguntavam aos visitantes o que mais tinham gostado na exposição.

Foram abordados 13 grupos familiares, entre os quais houve três recusas, sendo duas justificadas: em uma, a criança não quis colocar a câmera; em outra, o responsável explicou que fariam a visitação de forma rápida. Assim, ao todo, dez grupos participaram da pesquisa. Como mostra o Quadro 1, reunidos, os grupos totalizam 31 sujeitos, sendo 16 adultos (nove mulheres e sete homens) e 15 crianças (nove meninas e seis meninos). Quanto aos vínculos de parentesco em relação às crianças, havia mães, pais, tios, tias, primos, avôs e avós. Com exceção do grupo 1 e das crianças do grupo 10, os demais participantes residem na Colômbia; a maioria (seis grupos) em Bogotá, onde o museu se localiza. O tempo médio de visitação foi de 43 minutos.

**Quadro 1:** : Dados das famílias e tempo de visitação

Grupo	Adultos (Gênero, ID e idade)	Crianças (Gênero, ID e idade)	Cidade/País	Tempo de visitação
1	2 F (A1:39; A2: 39)	1 F (C1: 11); 1 M (C2: 6)	Santo Domingo-República Dominicana	00:23:00
2	2 F (A1: 25; A2: 47); 1 M (A3: 30)	1 F (C1: 7); 1 M (C2: 13)	Barranquilla-Colômbia	00:32:28
3	1 F (32)	1 F (10)	Bogotá-Colômbia	00:48:39
4	1 F (46)	1 M (11)	Bogotá-Colômbia	00:35:46
5	1 M (72)	1 M (8)	Pandi-Colômbia	01:01:00
6	1 F (A1: 46); 1 M (A2: 22)	2 M (C1: 12, C2: 13)	Bogotá-Colômbia	00:44:21
7	1 M (37)	1 F (8)	Bogotá-Colômbia	00:44:02
8	1 F (39); 1 M (38)	2 F (8 e 6)	Cota-Colômbia	00:58:20
9	1 M (46)	1 F (10)	Bogotá-Colômbia	00:56:47
10*	1 F (não informado); 1 M (24)	2 F (9; não informado)	Bogotá-Colômbia (adultos) Canadá (crianças)	00:27:37
**Total**	**9 F; 7 M**	**9 F; 6 M**		**07:12:00**

*As duas participantes que não informaram a idade chegaram após o início da visitação e se juntaram ao grupo.

Fonte: elaborado pelos autores.

A visita era realizada de forma livre, e, ao longo dela, em alguns momentos, todas as famílias contaram com o apoio de educadores(as). Na maioria desses momentos, o(a) educador(a) fez uma breve apresentação inicial e, em seguida, deixou os visitantes livres para fazer o percurso (G3, G4, G6, G7, G10). No entanto, também houve casos em que o(a) educador(a) apresentava uma estação, deixava os visitantes livres para explorá-lo, e depois apresentava a estação seguinte (G8, G9); interagia em momentos específicos ao longo da visita (G2, G5); ou acompanhava o grupo ao longo de todo o percurso (G1).

Os dados audiovisuais coletados foram analisados utilizando o *software* Dedoose, com base em protocolo de análise criado pelo grupo de pesquisa e já utilizado em estudos anteriores (Massarani et al., 2019a, 2019b). No Quadro 2 são especificadas as categorias analisadas neste artigo. O *software* utilizado possibilita quantificar as ocorrências e coocorrências^6^ dos códigos, bem como por quanto tempo cada código esteve presente ao longo das visitas.

**Quadro 2:** : Protocolo de análise

**1. Conversações**	
1.1 Conversas sobre temas de ciência	Diálogos sobre algum tema científico, discutem dilemas éticos e morais da ciência, impacto social da atividade científica, trazem dados ou conteúdos científicos, questões sobre a atuação profissional ou vida pessoal e aparência de cientistas. Entram aqui todos os campos de conhecimento científicos (ciências exatas e da terra, ciências biológicas, ciências humanas e sociais, ciências da saúde e ciências agrárias).
1.2 Conversas sobre a exposição e temática não científica	Diálogos sobre temáticas que são abordadas pela exposição, mas que não se referem a temas de ciência previstos na categoria anterior.
1.3 Conversas sobre a exposição (funcionamento, *design*, experiência museal)	Diálogo desencadeado sobre a exposição e/ou os módulos expositivos, seja sobre seu funcionamento, *design* e/ou experiência museal.
**2. Tipo de interação**	
2.1. Visitante-módulo expositivo	
2.1.1. Atividade interativa	A interação se dá por: imersão; experimentação; interação física (apertar botões, girar manivelas etc.) necessárias para a continuidade da narrativa/enredo/conteúdo do módulo; controle de variáveis e interferência no resultado final/produto do módulo; e/ou jogo.
2.1.2. Interação contemplativa	Contemplação, observação, visualização sem toque/manipulação de um módulo expositivo ou parte específica dela.
2.1.3. Leitura de painel/texto/foto explicativo	A interação se dá pela leitura em voz alta de textos (integrais ou parte) de placas informativas, painel, legenda, texto, charge dos módulos expositivos.
2.2. Visitante-mediador	Diálogos estabelecidos entre os visitantes e mediadores ou quando os visitantes escutam as orientações e informações do mediador, independentemente do conteúdo dessa conversa, podendo ser sobre temáticas da exposição, temas que a tangenciam, orientações sobre a visita e experiência museal ou não.
2.3. Visitante-visitante	Diálogos estabelecidos entre os visitantes, independentemente do seu conteúdo, ou seja, temáticas da exposição, temas que a tangenciam ou não. Pode ocorrer entre os visitantes de um grupo ou com visitantes que não pertencem ao mesmo grupo.
**3. Emoção**	
Expressão de algum sentimento durante a visita.	

Fonte: adaptado de Massarani et al. (2019a, 2019b).

É importante ressaltar que a categoria “emoções” reflete o ponto de vista dos pesquisadores. Não buscamos afirmar o que os visitantes de fato sentem, e sim refletir sobre a presença de expressões emocionais na relação entre sujeitos e exposição, a partir da observação dos pesquisadores. Em consonância com Rowe et al. (2023, p.79), buscamos observar “marcadores discursivos de emoção tais como entonação, expressão facial, gestos, palavras com conotação emocional, exclamações, risadas e expressões corporais”. Entretanto, foge ao escopo deste artigo o aprofundamento da reflexão sobre a dimensão emocional nas visitas a museus, tema complexo que esbarra em desafios metodológicos tais como a própria definição de emoção e a dificuldade em mensurá-las (Boehner et al., 2007; Boyd, Hugues, 2020; Falk, Gillespie, 2009; Massarani et al., 2022b; Myers Jr., Saunders, Birjulin, 2004).

## Resultados

Segundo informações fornecidas pelas famílias por meio de questionário, seis grupos têm o costume de visitar centros e museus de ciências uma vez ao ano (G1, G3, G5, G8, G9, G10) e dois visitam mais do que uma vez ao ano (G4, G6). Mas apenas um grupo (G8) já havia visitado o Maloka antes. Dentre as expectativas para a visita à exposição “Ciencia del amor y del perdón”, destaca-se a busca por aprendizado e conhecimento, citada por quatro grupos (G4, G7, G9, G10). Ao serem questionados, após a visita, sobre o que mais gostaram, o módulo “El miedo, emoción más antigua” foi o mais citado, sendo mencionado por pelo menos um indivíduo de cinco grupos diferentes (G1, G2, G5, G8, G9).

Na análise dos dados audiovisuais, foram aplicados, durante as sete horas e 12 minutos de visitação, 1.213 códigos, dos quais 1.130 compõem as categorias abordadas neste artigo. Conforme indicado na Tabela 1, os cinco códigos mais aplicados foram: conversas sobre a exposição e temática não científica (278), leitura de painel/texto/foto explicativo (200), atividade interativa (182), interação contemplativa (129) e conversas sobre a exposição (funcionamento, *design*, experiência museal) (125). Ao levarmos em consideração o fator tempo, os cinco códigos mais relevantes foram: interação visitante-visitante (presente em 80,7% do tempo total das visitas), atividade interativa (48,5%), interação visitante-mediador (36,3%), interação contemplativa (29%) e conversas sobre a exposição e temática não científica (17%). Notamos ainda que as conversas sobre temas de ciência, no que tange ao fator tempo, passam a ser o segundo tipo de conversa mais relevante, superando aquelas sobre a exposição (funcionamento, *design*, experiência museal).

**Tabela 1:** : Número de códigos aplicados e tempo na visita para cada tipo de conversa e interação analisado

Dimensão	Categoria	Número de códigos aplicados	Tempo na visita (%)*
Conversas	Exposição e temática não científica	278	17
	Exposição (funcionamento*, design*, experiência museal)	125	11,4
	Temas de ciência	61	14,6
Tipos de interações	Leitura	200	10,7
	Interação contemplativa	129	29,1
	Atividade interativa	182	48,5
	Visitante-visitante	46	80,8
	Visitante-mediador	42	36,3
Emoção		67	1,0
**Total**		**1.130**	

* Os dados não somam 100% porque a cada trecho de visita era possível aplicar mais de um código.

Fonte: elaborada pelos autores.

Os dados indicam que leitura de painel/texto/foto explicativo tem alto número de códigos aplicados (duzentos), mas porcentagem de tempo nas visitas relativamente baixa (10,7%). O mesmo ocorre com conversas sobre a exposição (funcionamento, *design*, experiência museal), que teve 125 códigos aplicados e 11,4% de tempo nas visitas. Esse padrão quantitativo indica interações frequentes, mas breves. Já as interações entre os sujeitos têm poucos códigos aplicados (42 entre mediador(a) e visitantes, e 46 dos visitantes entre si) e alta porcentagem de tempo (36,3% entre mediador(a) e visitantes e a expressiva marca de 80,8% dos visitantes entre si), indicando interações longas. As emoções, por sua vez, foram expressas pontualmente (67 episódios, em apenas 1% do tempo total das visitas).

Assim, os sujeitos, em geral, interagiram com a exposição tanto de forma ativa quanto contemplativa, trocando impressões com os seus familiares e com os(as) mediadores(as), e não de forma individualizada e introspectiva, inclusive decidindo coletivamente o percurso a ser feito.^7^ As conversas sobre ciências, por sua vez, também se caracterizaram por episódios longos, e, apesar de a porcentagem de tempo ser relativamente baixa no universo total da pesquisa (14,6%), ao levarmos em consideração apenas as conversas, elas são o segundo tipo mais relevante no fator tempo.

Em termos de coocorrência, as cinco mais relevantes estão indicadas na Tabela 2. Destaca-se a elevada coocorrência entre conversas sobre a exposição e temática não científica e atividade interativa, o que sugere que as atividades interativas propostas estimularam diálogos sobre o tema da exposição. Mas também foram relevantes os casos em que essas conversas estiveram associadas com a contemplação e a leitura de textos. Contemplação e realização de atividades interativas ocorreram juntas setenta vezes, sugerindo que, enquanto um visitante interagia ativamente com o aparato, os(as) demais não necessariamente o faziam junto, por vezes ficando contemplando a interação feita.

**Tabela 2:** : Coocorrências mais relevantes

Códigos em coocorrências		Número de coocorrências
Conversas sobre a exposição e temática não científica	Atividade interativa	109
Atividade interativa	Interação contemplativa	70
Conversas sobre a exposição e temática não científica	Interação contemplativa	67
Atividade interativa	Leitura de painel/texto explicativo	61

Fonte: elaborada pelos autores.

No que tange à interatividade emocional, é necessário apontar que os indicativos corporais e verbais de emoções foram pouco expressivos (67 ocorrências, 1% do tempo das visitas), constituindo-se principalmente em momentos de diversão, caracterizados por risadas ocorridas na interação entre os diferentes membros da família ou deles com o(a) educador(a). Assim, ainda que as famílias tenham refletido e conversado sobre emoções, demonstrando interatividade mental, os dados da pesquisa não nos permitem afirmar que a interatividade emocional tenha sido igualmente relevante.

A título de exemplo, o grupo 4, na estação “La alegría le da sabor a la vida”, diverte-se ao tirar as *selfies*: C1^8^ entra na estação, e A1 já havia tirado a foto.

C1: Estamos processando sua foto [leitura]. A1 e C1: [risos].

C1: Praia?

A1: Não, festa [selecionado o filtro]. [C1 e A1 riem da foto].

A1: Agora você.

C1: Festa, festa, festa. [A1 e C1 riem da foto].

O grupo 8, por sua vez, se diverte, junto com o(a) educador(a), ao interagir com o aparato da estação “La ira es un fuego interior que nos devora”, que convida a extravasar a raiva por meio de gritos:

A1: [grita muito alto]. [A1, A2, C1, C2 e E1 riem].

A2: Parabéns, você tem muita raiva. Agora aqui [rindo, indica outro lugar].

A1: [grita]. [A2 ri].

A2: Agora vocês [aponta para C1 e C2]. C1: [grita baixo]. [A1 e A2 riem].

A seguir, apresentamos alguns trechos categorizados como “conversas sobre temas de ciências” ou “conversas sobre a exposição e temática não científica”. Assim, inclui tanto diálogos sobre conteúdo científico como trechos relativos ao conteúdo expositivo, mas sem teor científico. Por meio desses exemplos, escolhidos por serem considerados emblemáticos do que observamos no *corpus* da pesquisa, analisaremos como se deu a interação entre os diferentes sujeitos envolvidos nas visitas, no que tange à abordagem dos temas científicos e não científicos apresentados na exposição.

Em interação do grupo 1 com a estação “El miedo, emoción más antigua”, o(a) educador(a) orienta sobre o uso do aparato, por meio de um direcionamento: “Agora coloca um medo. O nome de um medo que você tem”. Em seguida, o visitante adulto(a) faz uma ponte entre educador e criança, transformando o direcionamento em uma pergunta: “Qual é o nome de um medo que você tem?”. O diálogo continua entre os três, sendo a resposta fornecida, ao final, pelo(a) visitante adulto(a): C1: “Um nome?”. E1: “Um nome, sim. Pode ser a escuridão, cair, palhaços”. A1: “Ai, meu Deus! Avião, barata [risos]”. A proposta do aparato, bem como a pergunta feita, estimulam a autorreflexão sobre o medo.

Já em episódio ocorrido no grupo 8, enquanto interagia com a estação “La ira es un fuego interior que nos devora”, também existe formulação de perguntas sobre o conteúdo abordado, que, nesse caso, é feita pelo(a) educador(a): “Este módulo trata da ira. A ira é boa para muitas coisas. Para que serve a ira?”. Diferentemente do exemplo anterior, aqui, quem responde é uma das crianças, (C1): “Soltar todas as emoções que temos”. O diálogo continua, com a interação de diferentes membros do grupo entre si, com o(a) educador(a) e com o aparato:

A1: Muito bem.

A2: A ira é minha [emoção] favorita.

E1: Para soltar todas as coisas reprimidas. O que vão fazer aqui? Vem aqui onde vai gritar, coloca suas mãos aqui e grita até que acendam os níveis de raiva. [indica o local].

C1: Você grita [fala para A2].

A2: [grita].

C1: [grita].

A2: Faltou esse [indica o marcador luminoso].

C1: [grita de novo].

A2: Muito bem, agora [C2, nome da criança removido].

A1: [grita de novo].

C1: [grita de novo].

C2: Agora você.

A2: Não, não, agora você.

C1: [grita de novo].

A2: [C2, nome da criança removido], agora você.

C2: [está sentada no chão e se recusa a gritar].

C1 e A2: [gritam].

C1: [grita alto].

[A1 e A2 riem]

A2: Pronto, agora falta [C2, nome da criança removido].

C2: [permanece sentada e se recusa a gritar].

A2: Bem, agora vamos.

Nota-se que o diálogo inclui o direcionamento do(a) educador(a) sobre o uso do aparato, com o qual dois adultos e uma criança interagem, enquanto a outra criança não demonstra interesse na interação.

Em interação do grupo 9 com a estação “La indignación es una emoción que transforma”, a criança verbaliza uma dúvida: “Indig... [lê com certa dificuldade] O que é isso?”. O(a) visitante adulto(a) responde que é “como reagimos contra algo”. O(a) educador(a) complementa: “Bem, é como ira, mas causada por algo desconhecido, ou por algum problema”. Assim, há uma colaboração entre adultos em prol do aprendizado e fruição da criança. Na continuidade do diálogo, durante o qual o grupo interage com o aparato no qual todos são convidados a qualificar, de um a cinco, o quão indignantes consideram determinadas atitudes, há formulação de perguntas por parte do(a) educador(a), voltadas tanto para o uso do aparato quanto para a reflexão sobre a indignação. Todos os visitantes do grupo fornecem respostas a(o) educador(a) e interagem com o aparato:

A1: Olha as crianças [aponta para a tela].

E1: O que isso te causa?

C1: Tristeza e raiva.

E1: Exatamente. [Classifica] de um a cinco, sendo cinco a que mais raiva ou tristeza te causa. Pronto? Maltrato a animal, de um a cinco, quanto te indigna?

[C1 bate forte em um dos botões].

E1: Você, pai.

A2: Aqui? [indica o botão 5].

E1: Sim, cinco. Pronto. Agora vandalismo.

C1: Três.

E1: Três? Quatro [E1 aperta o 4]. Dano ambiental.

A1: Esse é cinco.

C1: Quatro.

E1: Maltrato à mulher. Ai! Esse é cinco. Deixa eu ver. O abandono infantil. O mais indignado fui eu?

A seguir, trazemos dois episódios no qual o(a) educador(a), além de orientar sobre o funcionamento do aparato e formular perguntas, também fornece informações científicas. O primeiro ocorreu no grupo 8, enquanto interagia com a estação “La tristeza acerca a los otros”. Já o segundo foi no grupo 3, em interação com a estação “¿Los animales sienten emociones?”. Em ambos os casos, todos os visitantes interagem com as perguntas.

E1: Esse é um módulo sobre a tristeza. Por que acham que a tristeza é importante?

A1: Para reconhecer o que é a alegria. [A2 ri].

E1: Sim, sim.

A1: Se não se sente triste, não sabe quando está alegre.

E1: O que acha? [aponta para C2]. Por que acha que a tristeza é importante? O que pensa? Ou não gosta da tristeza?

C2: Que está tudo bem.

E1: Tudo bem estar triste, certo? De vez em quando, tudo bem estar triste. Vou te contar algo curioso. A tristeza é importante porque nos ajuda a nos reconhecermos como humanos e que temos vínculos. Vocês têm [vínculos] com seus pais?

C1 e C2: Sim.

E1: E seus pais têm vínculos com vocês. Quando lhes acontece algo, vocês ficam tristes? [C1 balança a cabeça em afirmação].

E1: Provavelmente, sim. Se acontece algo ruim, vai chorar “ah mamãe, papai” [imita elas chorando]. Claro que sim. Então a tristeza nos lembra daqueles com os quais temos vínculos ou afetos e gera um processo na nossa cabeça muito interessante. Normalmente, quando estamos tristes, o que fazemos? Vocês estão tristes com algo em particular? [pergunta para C1 e C2].

C1: Os filmes.

E1: Sim. O que acontece nos filmes? Colocam canções tristes, e isso tem um porquê, quando estamos tristes escutamos músicas tristes. Mas por que escutar música triste se está triste? [C1 ri].

E1: Porque a música triste nos faz chorar, e chorar é um processo muito importante para a tristeza. Porque chorando nos sentimos melhor. Quando caem o que fazem? [pergunta para C1 e C2].

C1 e C2: Chorar. [A2 ri].

E1: E quando choram muito se sentem melhor? “Já não aguento mais chorar!” [imita uma pessoa exausta de tanto chorar] [C1, C2 e A1 riem].

E1: Bem, aqui encontrarão canções tristes, toca e escolhe uma canção. [indica a *jukebok* com as canções].

E1: Aqui fala um pouquinho sobre os animais e suas principais emoções: alegria e tristeza. Será que os cachorrinhos sentem alegria? [E1 utiliza a tela, C1 e A1 observam] [A1 olha para C1].

C1: Acho que sim.

A1: Sim.

E1: Sim? E por que acham isso? Posso lhes mostrar algo?

C1: Sim. [inaudível].

E1: A maioria dos animais que sentem emoções são mamíferos. O que acham que é isso? [E1 coloca um som de tristeza, feito por um mamífero].

C1: Tristeza.

E1: Pronto? Se querem, podem colocar aqui outros sons de animaizinhos [inaudível] [E1 se afasta do módulo, deixando C1 e A1 sozinhos].

A1: Vamos ver algo mais alegre [A1 utiliza o monitor *touchscreen*]. [leitura] Um leopardo ou um passarinho? Esse jovem orangotango vai ajudar o passarinho? ... O passarinho está esperando inquieto que chegue sua mãe com algo para comer. Esse jovem orangotango vai ajudar o passarinho? O que acha que vai acontecer? [continua com leitura silenciosa, acompanhando o texto com o dedo, e em seguida retoma a leitura em voz alta]. Ele o fez! Ajudou o passarinho. [C1 e A1 mudam de história, utilizando a tela, e retomam a leitura]. Em algum lugar tranquilo um encontro terrível está prestes a acontecer. [leitura]. Babuíno *versus* leopardo [indica na tela]. O felino sabe que com um babuíno é melhor não se meter. [fim da leitura]. Fugiu [indica na tela].

C1: Ahh, estão expressando emoções. [A1 e C1 ficam mexendo na tela].

A1: Vamos.

C1: Sim.

No caso supracitado, em dado momento, o(a) educador(a) deixa o grupo sozinho, o(a) visitante adulto(a) passa a fazer a mediação entre criança e aparato, por meio da leitura, e a criança faz uma reflexão (uma inferência de que os animais estão demonstrando emoções). Esse foi o padrão de interação quando o(a) educador(a) estava ausente, conforme podemos observar também no exemplo a seguir. Em interação do grupo 7 com a estação “La tristeza acerca los otros”, a família interage com o aparato utilizando-se da leitura, e a criança traça reflexões sobre o que a deixa triste:

A1: A *juke-box* da tristeza. [Leitura:] Que canção te deixa triste?

C1: [seleciona uma música].

A1: Essa?

C1: Sim, essa. Não, essa canção não me deixa triste. [C1 para a música e A1 seleciona outra].

C1: Essa me deixa triste. Voltar? [para a música, volta para tela inicial e seleciona outra]. Não me deixa triste. [muda a música]. Essa é a minha favorita [canta por um tempinho e muda de música]. Essa eu gosto.

A1: Isso te deixa triste?

C1: Quando minha vozinha partiu.

A1: Sim. [C1 cantarola].

A1: Seguimos.

Os dados indicam que as interações do(a) educador(a) com a família que se desdobram em conversas sobre temas de ciências ou sobre o conteúdo da exposição geralmente ocorrem por meio de indagações realizadas por esse profissional, que recebe respostas de adultos e/ou crianças. Os visitantes adultos, por sua vez, contribuem com o(a) educador(a) fazendo uma ponte entre ele(a) e a(s) criança(s). Já quando não há a presença de educadores, podemos dizer que o visitante adulto passa a exercer o papel da mediação humana, orientando a(s) criança(s). Assim, as interações entre adultos e crianças do grupo familiar são em geral relevantes, com ou sem a presença de educador. Nesse segundo caso, é comum que os visitantes recorram à leitura breve e parcial para compreender o funcionamento de aparatos, as atividades propostas e/ou o conteúdo abordado.

As conversas podem envolver a explicação do funcionamento do aparato, mas não se limitam a ela, sendo provocadas reflexões e conversas para além da manipulação dos totens. Assim, são interações nas quais adultos fazem perguntas ou direcionamentos que provocam o grupo a refletir sobre suas emoções, sendo essas reflexões, em alguns casos, necessárias para a tomada de decisão sobre a ação a ser realizada nos totens.

## Discussão

O alto índice de interações visitante-visitante observado na presente análise era esperado, uma vez se tratarem de famílias, e é consonante com dados encontrados em outros estudos, nos quais elas também se mostraram importantes para a construção de significado (Massarani et al., 2019b, 2022a; Shaby, Assaraf, Tal, 2019). Isso indica a relevância das visitas como um momento de lazer e interação no qual o aprendizado em família ocorre de forma colaborativa (Ash, 2003; Callanan, Cervantes, Loomis, 2011; Crowley, Pierroux, Knutson, 2014; Ellenbogen, 2002; Pattison, Rubin, Wright, 2017; Shaby, Assaraf, Tal, 2019).

Crowley, Pierroux e Knutson (2014) apontam que o papel dos familiares adultos pode variar segundo a agenda do grupo, ora deixando as crianças mais livres e se mantendo em segundo plano, menos ativos; ora se envolvendo mais nas atividades. No nosso estudo, eles se mostraram importantes mediadores entre as crianças e a exposição, fazendo perguntas, fornecendo explicações, tirando dúvidas, sugerindo ações, contribuindo para a superação de dificuldades e estimulando uma interação mais aprofundada. Segundo Ash (2003, p. 142), uma vez que os diferentes membros da família têm trajetórias, objetivos, conhecimentos prévios e habilidades específicos, “o conhecimento está distribuído em profundidade e temáticas”, o que “permite que os membros da família desenvolvam múltiplas formas de dialogar sobre biologia,^9^ usando suas áreas de interesse como foco”. Assim, “a exposição em si funciona como a fonte do conteúdo, mas ele é interpretado de forma diferente por cada membro da família, a partir de sua própria compreensão” sobre ciência (p.148), o que possibilita que surjam o que a autora chama de “diálogos investigativos”, nos quais um grupo social (no caso, a família) se engaja colaborativamente na(s) atividades(s). Assim, para ela, em uma visita familiar a um museu, o aprendizado pode ser promovido na medida em que os diferentes membros podem brincar, conversar e aprender juntos, e os visitantes adultos eventualmente se tornam mediadores(as) do processo de aprendizado das crianças. Sobre a realização de leituras pelas famílias, os dados deste estudo corroboram pesquisas anteriores que indicam que os textos presentes nas exposições são um importante suporte que os visitantes utilizam para encontrar informações e sanar dúvidas, por meio de leituras parciais (Massarani et al., 2021a, 2021b; McManus, 1989; Rocha et al., 2021; Rodrigues, Afonso, 2015).

Além da atuação dos familiares adultos, no presente estudo, os educadores da instituição também tiveram papel importante para a fruição dos grupos. Eles atuaram como comunicadores da proposta expográfica, estimulando a reflexão sobre as emoções, introduzindo conhecimentos novos e promovendo diálogos e aprendizado sobre ciências. Ainda que sua presença não deva ser imprescindível para a fruição das exposições, a mediação humana é uma característica marcante dos chamados museus de ciências de terceira geração (Cazelli, Marandino, Studart, 2003). Podendo ser exercida por diferentes atores, tais como curadores e educadores, esse tipo de mediação possibilita um direcionamento do processo de aprendizagem científica, aproximando os públicos da narrativa expográfica proposta (Cazelli, Marandino, Studart, 2003). Além disso, permite que a comunicação entre instituição e públicos se torne dialógica, considerando as especificidades dos visitantes, em vez de tratá-los como uma “audiência indiferenciada e passiva” (Rodari, Merzagora, 2007, p.9). Como apontam Pavão e Leitão (2007), o mediador exerce o papel de provocador e problematizador, estimulando a discussão, o levantamento de hipóteses e a busca por explicações e interpretações dos fenômenos e das ideias científicas abordadas na exposição. Assim, os educadores podem executar um papel importante na comunicação da instituição com os seus públicos (Rodari, Merzagora, 2007) e no estímulo a trocas, interlocução de saberes e construção de conhecimento (Pavão, Leitão, 2007).

Neste estudo de caso, consideramos que a função dos educadores na explicação das emoções e do seu papel nas relações humanas, em uma situação de superação de conflito armado que permeia a sociedade em diferentes escalas, pode ser uma contribuição importante para a busca da reconciliação socioemocional, abordada a nível individual e que “implica a capacidade de gerir as emoções e crenças ativadas em uma pessoa quando ela imagina ou tem contato com indivíduos que considerava adversários” (Alzate et al., 2018, p.82).

Ainda que se caracterizem por agendas distintas (institucional, no caso dos educadores, e pessoal, no caso dos familiares), as abordagens dos diferentes sujeitos adultos partícipes da visita tiveram em comum a perspectiva dialógica. Jant et al. (2014) apontam que a realização de perguntas abertas, por parte dos adultos, ao contribuir para o foco da atenção, pode facilitar o aprendizado das crianças. Para os autores, os diálogos possibilitam que o engajamento físico com os objetos se desdobre em uma experiência de aprendizado, inclusive facilitando a conexão entre conhecimentos prévios e novos. Em seu estudo de caso, eles notaram que, ainda que as crianças possam aprender por conta própria, as ações e conversas com outras pessoas influenciam bastante no que elas irão aprender, guardar, compartilhar e transferir da experiência. Além disso, em estudo de caso no contexto britânico, Studart (2005, p. 63) notou que as crianças, ao visitarem museus com seus familiares, “valorizam a atenção pessoal e dedicada que recebem de seus pais e parentes, bem como a possibilidade de poder perguntar tão logo não compreendam algo”.

Quanto à proposta expográfica, o fato de as atividades interativas terem coocorrido mais vezes com conversas sobre o tema da exposição do que com conversas sobre o funcionamento dos aparatos sugere que os módulos interativos, articulados à mediação humana realizada pelos educadores do museu e/ou pelos visitantes adultos, foram capazes de promover conversas sobre ciências e sobre o conteúdo expositivo. Assim, a interatividade, como um importante recurso para a fruição e o aprendizado (McManus, 1992), foi pensada além de uma perspectiva estritamente física, sendo alcançada interatividade mental (Wagensberg, 2005). A narrativa expositiva articula o debate científico sobre emoções com temas do cotidiano, e as diferentes estações propunham que os(as) visitantes interagissem fisicamente com os aparatos, mas, em grande parte das atividades propostas, essa interação física exigia reflexões e tomadas de decisão, buscando articular interatividades de tipo manual e mental. Além disso, alguns módulos, por meio da imersão, buscavam despertar emoções nos visitantes (interatividade emocional).

É interessante destacar ainda que, por meio de suas decisões na interação com os aparatos, são os visitantes que definem a experiência. Para Padilla (2001), essa se constitui em uma importante forma de promover a participação dos públicos. Consideramos que o fato de a exposição abordar temáticas associadas à superação das diferenças, que emergem em um contexto prolongado de guerra civil, reflete o interesse institucional em se apresentar como um museu de ciências atento a temas socialmente relevantes, buscando promover o debate sobre eles. Além disso, indica que a instituição se pauta pela compreensão de que a ciência e a sua representação em exposições não são neutras (Marandino, Contier, 2017), sendo atravessadas por sua dimensão política, ou seja, por dinâmicas de poder (Hodson, 2013; Macdonald, 1997; Macdonald; Silverstone, 1992).

## Considerações finais

Neste artigo, investigamos as visitas de dez famílias à exposição “Ciencia del amor y del perdón”, do centro de ciência Maloka. Os resultados indicaram que as visitas se constituíram como um momento de lazer, interação e aprendizado em família, sendo marcadas por interações frequentes e longas entre as famílias e os(as) educadores(as) da exposição e, principalmente, dos membros da família entre si, demonstrando a relevância do papel dos adultos para a fruição por parte das crianças. Tais interações envolveram principalmente conversas sobre a temática da exposição, de cunho científico ou não, entremeadas por interações contemplativas, atividades interativas e leituras breves. Os textos da exposição se mostraram recursos importantes, principalmente quando os(as) educadores(as) da instituição estavam ausentes, pois a família recorria a eles para buscar informações sobre as atividades propostas e o conteúdo científico abordado.

A relevância das conversas sobre o conteúdo da exposição durante as atividades interativas são indícios de que houve interatividade mental. Argumentamos que isso se tornou possível pela atuação dos adultos, mas também pela proposta expográfica, que estimulava a reflexão no âmbito da interação com os aparatos. Assim, acreditamos que a exposição tem o potencial de envolver as pessoas a partir de sua subjetividade e promover interações e reflexões sobre si mesmos e suas relações. Por outro lado, a interatividade emocional não emergiu nos dados, já que houve baixa expressividade da categoria “emoções”. Destacamos que a metodologia utilizada nos permitia acessar expressões verbais e corporais indicativas de emoções, mas outras metodologias, como entrevistas e autorrelatos dos visitantes, mostram-se importantes para aprofundar as reflexões sobre a interatividade emocional em museus.

Ainda no que tange às limitações da pesquisa, compreendemos que acessar as experiências dos visitantes é desafiador. Do ponto de vista metodológico, o uso das câmeras GoPro é interessante porque permite o acesso às experiências das famílias a partir do olhar das crianças e sem a presença dos pesquisadores durante as visitas, o que poderia modificar a dinâmica dos grupos familiares. Por outro lado, reconhecemos que, a partir do momento em que os participantes tomam ciência da realização da pesquisa e sabem que as visitas estão sendo gravadas, já há uma influência que possivelmente modifica em algum grau a dinâmica dos grupos. Ainda que compreendamos que toda pesquisa social está sujeita a esse tipo de mudança decorrente da necessária interlocução entre pesquisadores e pesquisados, salientamos a relevância de que sejam realizados estudos que aprofundem a compreensão da influência do uso das câmeras nesse tipo de pesquisa.

Consideramos que o estudo trouxe contributos importantes para a compreensão das especificidades das visitas em família e da atuação de educadores(as) museais em um contexto expográfico atravessado por questões sociais sensíveis. A continuidade das pesquisas mostra-se relevante, a fim de aprofundar o debate sobre o modo como os museus de ciências podem contribuir para a construção de diálogos na sociedade, em torno de situações complexas que emergem em diferentes escalas, quando uma sociedade enfrenta coletivamente um conflito interno prolongado, como é o caso da Colômbia.

## Data Availability

Não estão em repositório.

## NOTAS

1 Algumas instituições citadas por Giroto (2021) que tematizam as memórias relativas às ditaduras são o Museo de la Memoria y los Derechos Humanos, em Santiago (Chile); o Memorial da Resistência, em São Paulo (Brasil); o Espacio Memoria y Derechos Humanos, em Buenos Aires (Argentina); e o Sitio de Memoria e o Museo de la Memoria, ambos localizados em Montevidéu (Uruguai). Quanto àquelas que tematizam os conflitos armados da atualidade, o autor menciona o Centro de Memoria, Paz y Reconciliación, em Bogotá (Colômbia), e o Lugar de la Memoria, la Tolerancia y la Inclusión Social, em Lima (Peru).

2 A pesquisa “Olhar do visitante” como um todo, incluindo este artigo, foi aprovada pelo Comitê de Ética da Fundação Oswaldo Cruz (CAAE 10663419.0.0000.5241).

3 Esta e as demais citações de textos e conversas em língua estrangeira têm tradução livre.

4 Existe uma versão digital da exposição, disponível em: https://maloka.org/ciencia-del-amor-y-del-perdon/. Acesso em: 20 ago. 2024. Ainda que a exposição digital apresente algumas diferenças em relação à física, com o intuito de adaptá-la ao ambiente virtual, as informações disponíveis no *link* permitem a aproximação das propostas.

5 Alguns(mas) educadores(as) da instituição também contribuíram na abordagem dos grupos e aplicação do questionário.

6 Situações em que dois códigos são identificados simultaneamente.

7 Isso pode ser verificado nos exemplos de interações entre visitantes apresentados ao longo do artigo.

8 Ao longo de todo o artigo, para fins de apresentação dos dados, o código “C” se refere a visitante criança, “A”, a visitante adulto(a), e “E”, a educador(a). As numerações (C1, C2, A1, A2, por exemplo) têm por intuito distinguir entre diferentes membros de um mesmo grupo, utilizando como base a codificação apresentada no Quadro 1.

9 A autora analisou especificamente conversas sobre biologia, mas podemos ampliar a discussão para conversas sobre outras ciências, relacionadas à temática de cada exposição e/ou museu.

## References

[B1] ALZATE Mónica (2018). Dimensiones socioemocional e instrumental de la reconciliación social en el conflicto armado colombiano. Revista de Estudios Sociales.

[B2] ASH Doris (2003). Dialogic inquiry in life science conversations of family groups in a museum. Journal of Research in Science Teaching.

[B3] BOEHNER Kirsten (2007). How emotion is made and measured. International Journal of Human-Computer Studies.

[B4] BOYD Candice P., HUGUES Rachel (2020). Emotion and the contemporary museum: development of a geographically-informed approach to visitor evaluation.

[B5] CALLANAN Maureen, CERVANTES Christi, LOOMIS Molly (2011). Informal learning. Wiley Interdisciplinary Reviews Cognitive Science.

[B6] CAZELLI Sibele, MARANDINO Martha, STUDART Denise Coelho, Gouvêa Guaracira, Marandino Martha, Leal Maria Cristina (2003). Educação e museu: a construção social do caráter educativo dos museus de ciências.

[B7] COLOMBIA (2016). Acuerdo final para la terminación del conflicto y la construcción de una paz estable y duradera.

[B8] CROWLEY Kevin (2001). Shared scientific thinking in everyday parent-child activity. Science Education.

[B9] CROWLEY Kevin, PIERROUX Palmyre, KNUTSON Karen, Sawyer R. Keith (2014). The Cambridge handbook of the learning sciences.

[B10] ELLENBOGEN Kirsten M, Leinhardt Gaea, Crowley Kevin, Knutson Karen (2002). Learning conversations in museums.

[B11] ELLENBOGEN Kirsten M., LUKE Jessica, DIERKING Lynn D, Falk John Howard, Dierking Lynn Diane, Foutz Susan (2007). In principle, in practice: museums as learning institutions.

[B12] FALK John H., GILLESPIE Katie L. (2009). Investigating the role of emotion in science center visitor learning. Visitor Studies.

[B13] GALTUNG Johan (1996). Peace by peaceful means: peace and conflict, development and civilization.

[B14] GASPAR Alberto (1993). Museus e centros de ciências: conceituação e proposta de um referencial teórico.

[B15] GIROTO Ivo (2021). Museus de memória traumática na América Latina: uma leitura arquitetônica com base na emoção e na experiência. Arq.urb - Revista do programa de pós-graduação stricto sensu em arquitetura e urbanismo da Universidade São Judas Tadeu.

[B16] HODSON Derek (2013). Don't be nervous, don't be flustered, don't be scared: be prepared. Canadian Journal of Science, Mathematics and Technology Education.

[B17] JANT Erin A. (2014). Conversation and object manipulation influence children's learning in a museum. Child Development.

[B18] LUSKIN Fred (2002). Forgive for good: a proven prescription for health and happiness.

[B19] MACDONALD Sharon, Macdonald Sharon (1997). The Politics of display: museums, science, culture.

[B20] MACDONALD Sharon, SILVERSTONE Roger (1992). Science on display: the representation of scientific controversy in museum exhibitions. Public Understanding of Science.

[B21] MARANDINO Martha, CONTIER Djana (2017). Controvérsias, museus e exposições: será esse um bom momento para incrementar a relação entre museus e públicos?. Jornal Pensar a Educação em Pauta.

[B22] MARÍN Paola, ALZATE Gastón (2020). Corpos ausentes e armas fundidas: arte e necropolítica na Colômbia. Conception.

[B23] MASSARANI Luisa (2022). Experiências de aprendizagem em visita familiar à exposição "Quando nem tudo era gelo" do Museu Nacional. Ensaio: Pesquisa em Educação em Ciências.

[B24] MASSARANI Luisa (2022b). O papel das emoções na visita de adolescentes ao Aquário Marinho do Rio de Janeiro. Revista CTS.

[B25] MASSARANI Luisa (2021a). Da interação ao engajamento: um estudo da experiência de visita de adolescentes à exposição "Explora" no Pavilhão do Conhecimento, em Lisboa. Revista Portuguesa de Educação.

[B26] MASSARANI Luisa (2021b). A experiência interativa de famílias em um museu de ciências: um estudo no Museu de Ciência e Tecnologia de Porto Alegre. Investigações em Ensino de Ciências.

[B27] MASSARANI Luisa (2020). O olhar dos adolescentes em uma visita ao Museo Interactivo de Economía (MIDE), México. Revista Iberoamericana de Ciencia, Tecnología y Sociedad - CTS.

[B28] MASSARANI Luisa (2019a). La interactividad en los museos de ciencias, pivote entre expectativas y hechos empíricos: el caso del Centro Interactivo de Ciencia y Tecnología Abremate (Argentina). Ciência & Educação.

[B29] MASSARANI Luisa (2019b). A experiência de adolescentes ao visitar um museu de ciência: um estudo no Museu da Vida. Ensaio - Pesquisa em Educação em Ciências.

[B30] MCCLAIN Lucy R., ZIMMERMAN Heather Toomey (2019). Family connections to local science issues: how scientists use questions to engage families in personally relevant learning during science-themed workshops. International Journal of Science Education, Part B: Communication and Public Engagement.

[B31] MCCLAIN Lucy R., ZIMMERMAN Heather Toomey (2016). Memories on the trail: families connecting their prior informal learning experiences to the natural world during nature walks. Journal of Interpretation Research.

[B32] MCCLAIN Lucy R., ZIMMERMAN Heather Toomey (2014). Prior experiences shaping family science conversations at a nature center. Science Education.

[B33] MCMANUS Paulette M (1992). Topics in museums and science education. Studies in Science Education.

[B34] MCMANUS Paulette M (1989). Oh, yes, they do: how museum visitors read labels and interact with exhibit texts. Curator - The Museum Journal.

[B35] MYERS Olin Eugene, SAUNDERS Carol D., BIRJULIN Andrej A. (2004). Emotional dimensions of watching zoo animals: an experience sampling study building on insights from psychology. Curator.

[B36] PADILLA Jorge, Crestana Silverio (2001). Educação para a ciência: curso para treinamento em centros e museus de ciência.

[B37] PATTISON Scott, RUBIN Andree, WRIGHT Tracey (2017). Mathematics in informal learning environments: A summary of the literature (updated).

[B38] PAULA Lívia Mascarenhas (2017). Para além do apertar botões: a função social dos museus participativos de ciências.

[B39] PAVÃO Antonio Carlos, LEITÃO Angela (2007). Diálogos & ciência: mediação em museus e centros de ciência.

[B40] ROCHA Jéssica Norberto (2021). Um estudo sobre a leitura e a interação de adolescentes durante uma visita ao museu de ciências Universum, México.

[B41] RODARI Paola, MERZAGORA Matteo, Massarani Luisa, Rodari Paola, Merzagora Matteo (2007). Diálogos & ciência: mediação em museus e centros de ciência.

[B42] RODRIGUES Francisco E., AFONSO Ana Sofia (2015). Partilhar para aprender: um estudo sobre as interações dos alunos nos módulos de ótica de um museu de ciência. Alexandria - Revista de Educação em Ciência e Tecnologia.

[B43] ROWE Shawn (2023). Emotion in informal learning a mediated action: cultural, interpersonal and personal lenses. International Journal of Studies in Education and Science (IJSES).

[B44] SÁPIRAS Agnes (2007). Aprendizagem em museus: uma análise das visitas escolares no Museu Biológico do Instituto Butantan.

[B45] SHABY Neta, ASSARAF Orit Ben-Zvi, TAL Tali (2019). Engagement in a science museum: the role of social interactions. Visitors Studies.

[B46] STUDART Denise C (2005). Museus e famílias: percepções e comportamentos de crianças e seus familiares em exposições para o público infantil. História, Ciências, Saúde - Manguinhos.

[B47] VASCONCELLOS Camilo de Mello, Villaça Mariana, Schmiedecke Natália Ayo, Garcia Tânia Costa (2023). Políticas culturais na América Latina: entre conflitos e negociações.

[B48] VYGOTSKY Lev Semionovitch, Cole Michael (1978). Mind in society: development of higher psychological processes.

[B49] WAGENSBERG Jorge (2005). The "total" museum, a tool for social change. História, Ciências, Saúde - Manguinhos.

